# SF002-96-1, a new drimane sesquiterpene lactone from an *Aspergillus* species, inhibits survivin expression

**DOI:** 10.3762/bjoc.9.323

**Published:** 2013-12-13

**Authors:** Silke Felix, Louis P Sandjo, Till Opatz, Gerhard Erkel

**Affiliations:** 1 Institute of Biotechnology and Drug Research (IBWF), Erwin-Schrödinger-Straße 56, D-67663 Kaiserslautern, Germany; 2 Institute of Organic Chemistry, University of Mainz, Duesbergweg 10–14, D-55128 Mainz, Germany,; 3 Department of Molecular Biotechnology and Systems Biology, University of Kaiserslautern, Paul-Ehrlich-Straße 23, D-67663 Kaiserslautern, Germany

**Keywords:** apoptosis, inhibitor, natural products, secondary metabolite, structure elucidation, survivin

## Abstract

Survivin, a member of the IAP (inhibitor of apoptosis) gene family, is overexpressed in virtually all human cancers and is functionally involved in the inhibition of apoptosis, regulation of cell proliferation, metastasis and resistance to therapy. Because of its upregulation in malignancy, survivin has currently attracting considerable interest as a new target for anticancer therapy. In a screening of approximately 200 strains of imperfect fungi for the production of inhibitors of survivin promoter activity, a new drimane sesquiterpene lactone, SF002-96-1, was isolated from fermentations of an *Aspergillus* species*.* The compound inhibited survivin promoter activity in transiently transfected Colo 320 cells in a dose dependent manner with IC_50_ values of 3.42 µM (1.3 µg/mL). Moreover, it also reduced mRNA levels and protein synthesis of survivin and triggered apoptosis.

## Introduction

Survivin, a member of the inhibitor of apoptosis (IAP) protein family is one of the most prominent cancer-associated proteins identified to date, being upregulated in almost all human cancer types while usually undetectable in normal terminally differentiated adult tissues [1–2]. It plays a key role in both apoptosis and control of cell cycle progression and high expression of survivin in tumors correlates with increased drug resistance, an accelerated rate of recurrence and poor patient survival [3–4]. Survivin blocks apoptosis by protein–protein interactions via its characteristic baculovirus IAP repeat domain (BIR) resulting in the formation of a complex with hepatitis B X-interacting protein bound to caspase 9 that prevents recruitment of apoptotic protease activating factor 1 (Apaf-1) to the apoptosome and enhancement of X-linked inhibitor of apoptosis (XIAP)-mediated caspase 3 and 9 inhibition [5–6]. In addition, survivin plays an important role in controlling cell division. In normal differentiated tissues the expression of survivin is cell cycle-regulated with an expression maximum in G2/M phase [7]. During mitosis, survivin is associated in the chromosomal passenger complex (CPC), a multi-protein complex, including the proteins Aurora B kinase, inner centromere protein (INCENP) and Borealin/Dasra-B. In this complex, survivin promotes mitosis by mediating the segregation of sister chromatids, stabilization of microtubules in late mitosis and acting as an interphase between the centromere/central spindle and the CPC [8–9]. Furthermore, survivin shuttles between the nucleus and the cytoplasm where the dynamic intracellular localization is regulated by active nuclear export. Nuclear survivin has been described to control cell division whereas cytoplasmic survivin plays a role in cytoprotection. Several studies have shown a correlation between overexpression of cytoplasmic survivin and a poor outcome in various types of cancer although nuclear survivin has also been associated with a poor prognosis in cancer patients [10–12]. Given the critical role of survivin in cancer progression and treatment resistance, survivin has been emerged as an attractive target for new anticancer therapeutics [13]. In a search for new inhibitors of survivin expression from natural sources, we found that cultures of *Aspergillus* sp. strain IBWF002-96 produced a new drimane sesquiterpene lactone, SF002-96-1, with inhibitory activity on survivin promoter activity in transiently transfected Colo 320 cells. In the current study, the fermentation, structure elucidation, and some biological properties of the compound are described.

## Results and Discussion

### Identification and structure elucidation

SF002-96-1 was obtained by a bio-guided isolation procedure as a colourless oil. It gave a pseudo-molecular ion of *m*/*z* 403.2090 (calcd for [C_21_H_32_O_6_ + Na]^+^, 403.2097) in its HR-ESI mass spectrum. The composition accounted for six double bond equivalents and a characteristic absorption band for α,β-unsaturated γ-lactones was revealed in the UV spectrum at 207 nm. This was supported by IR absorption bands at 1757 and 1632 cm^−1^. Apart from that, absorption bands of an OH-group (3411 cm^−1^) and ester function (1737 cm^−1^) were revealed in the IR spectrum of the compound. The NMR spectra disclosed four CH_3_ groups (δ 0.97/32.6, 1.15/24.9, 1.04/12.8, and 0.87/14.2), seven CH_2_ groups (δ 1.28/31.9, 1.30/23.0, 1.59/25.1, 2.32/35.1, (1.34, 1.37)/42.7, (1.57, 1.66)/28.2, and (4.23, 4.48)/77.4), four CH groups (δ 1.94/45.7, 4.02/70.7, 5.72/67.0, and 6.57/133.5) and six quaternary carbons (δ 34.0, 44.7, 77.0, 133.8, 169.8, and 173.5, Table 1).

**Table 1:** ^1^H (600 MHz) and ^13^C NMR (150 MHz) data of SF002-96-1 in CD_3_CN.

Position	δ_H_ (multiplicity, coupling constant)	δ_C_

1	4.02 (dd, 4.4, 11.9 Hz, 1H)	70.7
2	1.57 (m, 1H), 1.66 (m, 1H)	28.2
3	1.34 (m, 1H), 1.37(m, 1H)	42.7
4	–	34.0
5	1.94 (overlapped with the solvent peak)	45.7
6	5.72 (dd, 4.0, 4.7 Hz, 1H)	67.0
7	6.57 (d, 4.0 Hz, 1H)	133.5
8	–	133.8
9	–	77.0
10	–	44.7
11	4.23 (d, 10.5 Hz, 1H), 4.48 (d, 10.5 Hz, 1H)	77.4
12	–	169.8
13	1.15 (s, 3H)	24.9
14	0.97 (s, 3H)	32.6
15	1.04 (s, 3H)	12.8
1′	–	173.5
2′	2.32 (m, 2H)	35.1
3′	1.59 (m, 2H)	25.1
4′	1.30 (m, 2H)	23.0
5′	1.28 (m, 2H)	31.9
6′	0.87 (t, 7.0 Hz, 3H)	14.2
OH-1	2.96 (br s, 1H)	–
OH-9	3.76 (br s, 1H)	–

**Figure 1:**
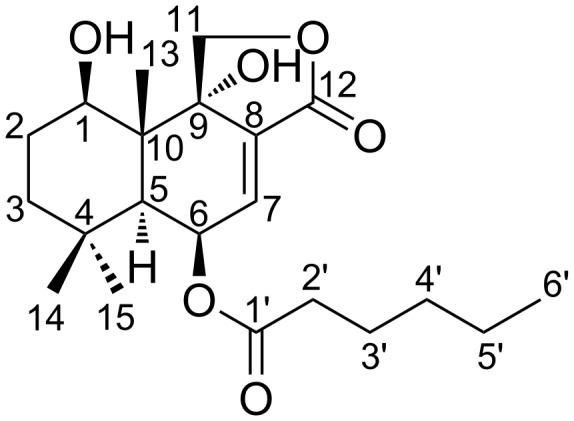
Structure of SF002-96-1.

The proton signal at δ 4.02 showed a COSY contact with methylene protons at δ 1.57 and 1.66 while the latter presented the same correlation with methylene protons at δ 1.34 and 1.37. The protons of two geminal methyl groups (δ 0.97, 1.15) displayed HBMC correlations with the methine carbon at δ 45.7, a quaternary carbon at δ 34.0 and the methylene carbon at δ 42.7 bearing protons at δ 1.34 and 1.37. Furthermore, a methyl group at δ 1.04 correlated with the oxymethine at δ 70.7, a methine at δ 45.7, and two quaternary carbons at δ 44.6 and 77.0. The correlations from the COSY spectrum between the proton signals at δ 1.94 and 5.72, as well as between those at δ 5.72 and 6.57 in conjunction with HMBC correlations observed between the proton signal at δ 6.57 and quaternary carbons at δ 77.0 and 133.8 suggested a decalin core for SF002-96-1. Further HMBC correlations disclosed between the signals at δ 6.57 and 169.8 as well as between the methylene protons at δ 4.23 and 4.48 and the carbons at δ 44.6, 77.0, 133.8, and 169.8 suggested SF002-96-1 to be a drimane sesquiterpene (Figure 1).

**Figure 2:**
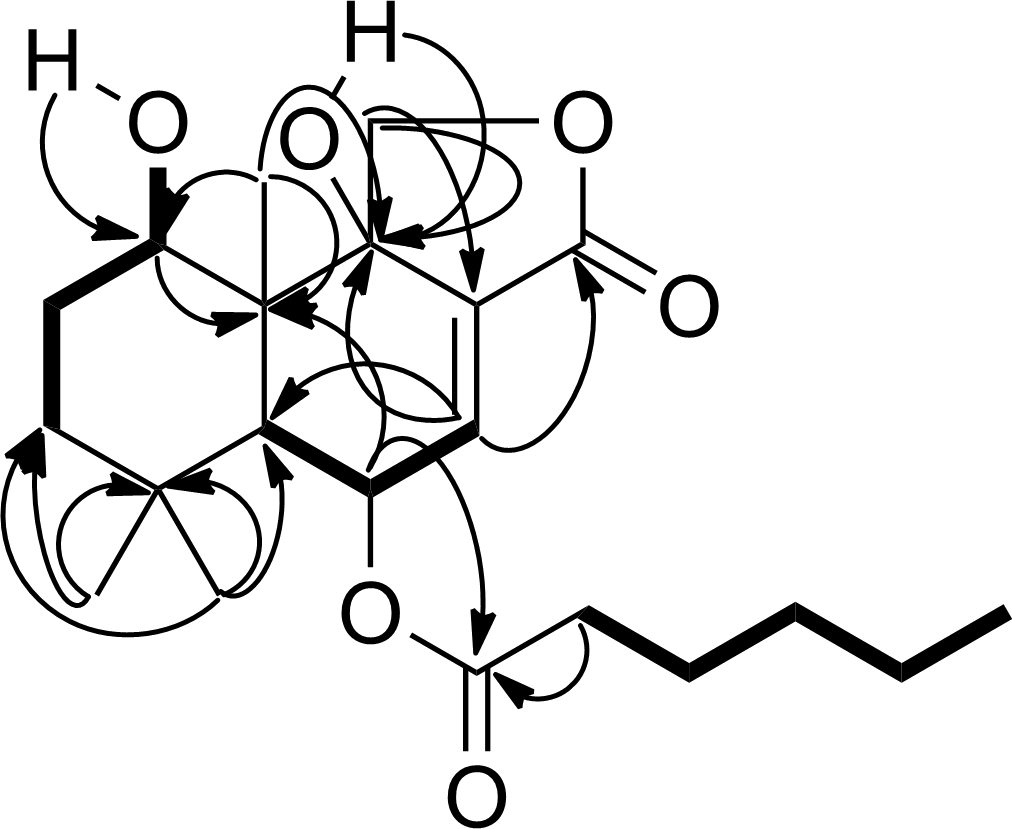
COSY (bold) and HMBC (arrow) correlations of SF002-96-1.

The proton signals of the methyl group (δ 0.87), four methylene groups (δ 1.28, 1.30, 1.59 and 2.32) and a carbonyl group (δ 173.5) constituted an *n*-hexanoyl chain. This conclusion was supported by correlations found in the COSY and HMBC spectra (Figure 2).

**Figure 3:**
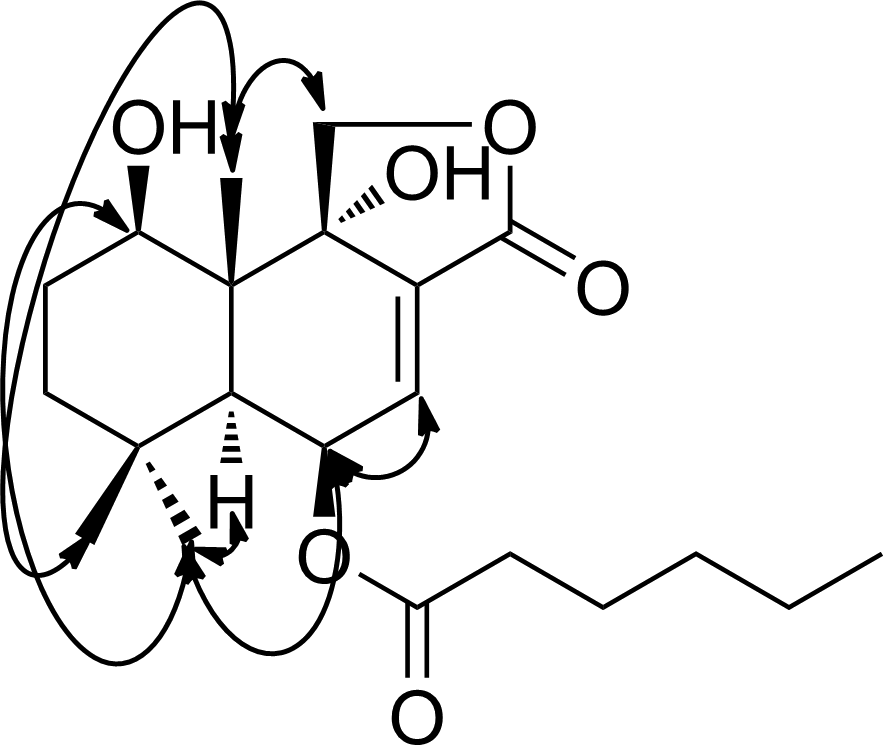
NOESY correlations of SF002-96-1.

The latter NMR data indicated that the side chain was attached to the allylic oxymethine in the drimane scaffold based on a HMBC contact between the proton signal at δ 5.72 and the carbonyl of the side chain (δ 173.5). The relative configuration was deduced from NOESY data. Thus, protons of a methyl group at δ 1.04 showed NOE correlations with those of one of the geminal methyl groups (δ 1.15) and one of the oxymethylene protons (δ 4.48) of the lactone ring (Figure 3).

Besides, the protons of the second geminal methyl group (δ 0.97) showed spatial correlations with a methine proton at δ 1.94 and two downfield protons at δ 4.02 and 5.72. Based on the absolute configuration reported for drimane sesquiterpenes [14–15], the *trans*-decalin core of SF002-96-1 was tentatively assigned the configurations *R*, *S*, *R*, *S*, and *R*, at C-1, C-5, C-6, C-9, and C-10, respectively. The foregoing data led to identify SF002-96-1 as (5*R*,5a*S*,9*R*,9aR,9bS)-9,9b-dihydroxy-6,6,9a-trimethyl-3-oxo-1,3,5,5a,6,7,8,9,9a,9b-decahydronaphtho[1,2-*c*]furan-5-yl hexanoate.

### Biological activity

**Figure 4:**
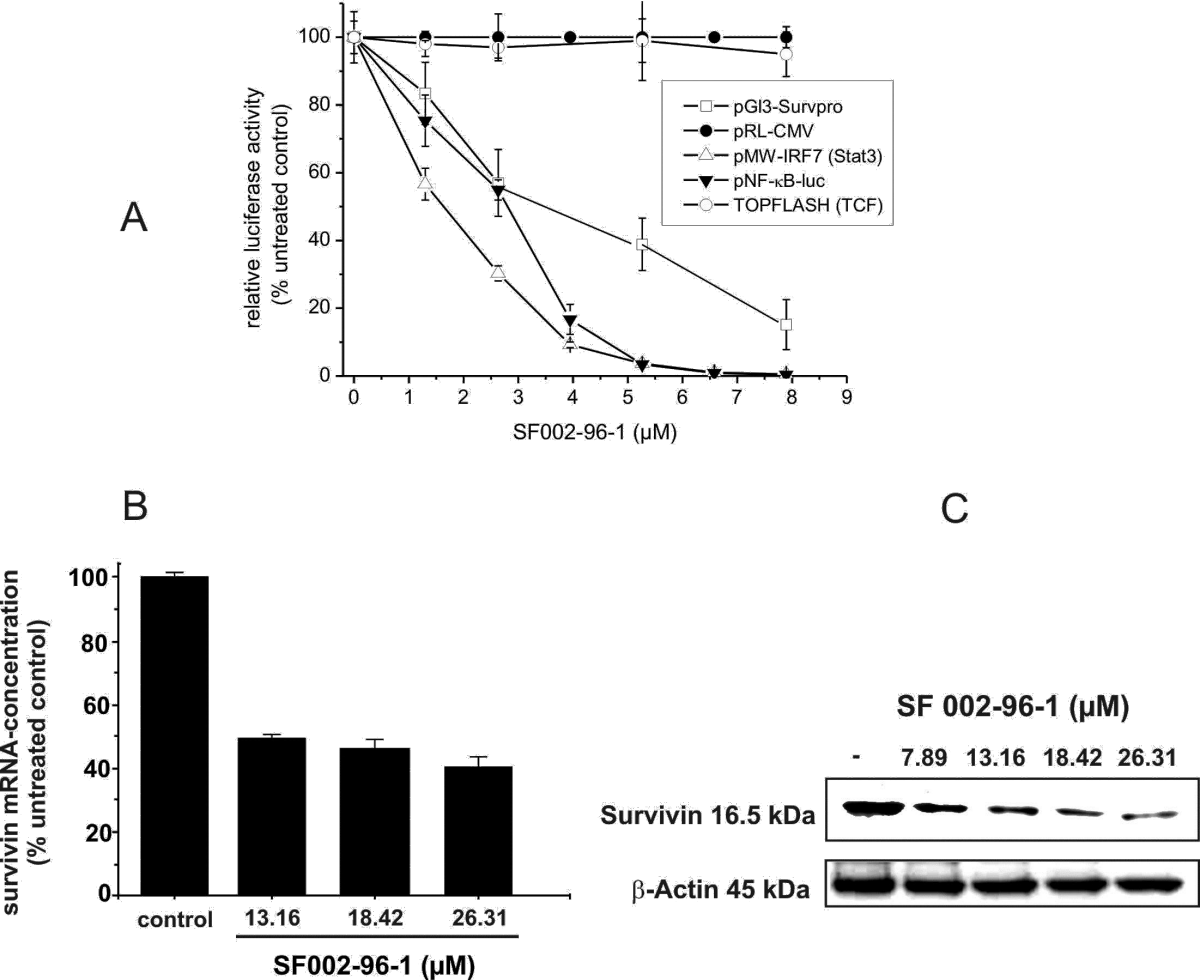
Effect of SF002-96-1 on survivin promoter activity, survivin mRNA levels and expression. (A) Colo 320 cells were transiently transfected with a human survivin-, CMV-, or β-catenin/TCF-dependent reporter construct for 24 h with or without test compound. Control (100%): untreated cells only. For NF-κB- and Stat3-driven reporter gene expression, cells were transfected with the indicated reporter gene construct and stimulated with 10 ng/mL TNF-α, 5 ng/mL IL-1β (for pNF-κB-luc) or 10 ng/mL IL-6 (for Stat3 dependent pMW-IRF7) with or without test compound for 24 h. Control: (100%): stimulation only. Results represent the mean ± SD of at least three independent experiments. The expression of the reporter gene was determined as described in the experimental section. (B) Effect on survivin mRNA levels. Colo 320 cells were treated with different concentrations of SF002-96-1 for 8 h. mRNA levels of survivin were measured by real-time PCR as described in the experimental section. Control (100%): untreated cells. Results represent the mean ± SD of at least three independent experiments. (C) Western blot analysis for survivin. Colo 320 cells were treated with different concentrations of SF002-96-1 for 8 h. Subsequently total cell extracts were prepared and analyzed by Western blot analyses. β-Actin was used as internal control.

For the identification of active secondary metabolites and the characterization of their influence on survivin expression, we employed a human survivin-promoter dependent transcriptional reporter in the transiently transfected human colorectal carcinoma cell line Colo 320. Due to the overexpression of survivin in many human cancers, including colon cancer, a strong constitutive luciferase activity could be observed 24 h after transfection caused by the binding of transcription factors to the regulatory sites of the survivin gene [16]. As shown in Figure 4A, SF002-96-1 inhibited survivin promoter activity in a dose dependent manner with an IC_50_ value of 3.42 µM (1.3 µg/mL), whereas the constitutive activity of the CMV promoter was not affected at concentrations up to 10.5 µM (4 µg/mL) indicating that the compound does not interfere with transcription in a general manner.

The expression of the survivin gene is regulated by a number of transcription factors including Stat3, NF-κB and the β-catenin activated T-cell factor (TCF) [17]. Constitutive activation of Stat3 by paracrine and autocrine mechanisms has been detected in diverse human cancer cell lines and tissues which contribute to oncogenesis by promoting cell proliferation and inhibiting apoptosis by increasing the expression of anti-apoptotic genes such as survivin [18]. We therefore investigated the effect of SF002-96-1 on Stat3-driven expression of the reporter gene luciferase in IL-6 stimulated Colo 320 cells. The compound strongly inhibited the Stat3-dependent luciferase expression with an IC_50_ value of 1.6 µM (0.6 µg/mL). In addition to Stat3, the response of the survivin promoter construct also depends on the transcription factor NF-κB [19] and as shown in Figure 4A, SF002-96-1 inhibited the inducible NF-κB-dependent reporter gene expression in Colo 320 cells with an IC_50_ value of 2.63 µM (1 µg/mL). Previous studies in colon cancer cells have shown that regulation of survivin expression is, in addition, TCF/β-catenin dependent mediated by three TCF/β-catenin consensus sequences within the survivin promoter [20]. Dysregulation of TCF/β-catenin dependent gene regulation originating from aberrantly stabilized β-catenin or mutations in the associated protein adenomatous polyposis coli (APC) results in the activation of target genes implicated in cell proliferation and transformation [21]. We therefore investigated the effect of SF002-96-1 on canonical Wnt signaling using a synthetic TCF-responsive reporter (TOPFLASH) in Colo 320 cells which contain a truncated APC protein [22] and therefore display high constitutive TCF/β-catenin transcription. Interestingly, SF002-96-1 showed no significant inhibition of the TCF/β-catenin dependent luciferase expression up to the highest concentration tested (8 µM).

To investigate the effect of the compound on the transcription of the survivin gene, quantitative real-time PCR experiments were performed with total RNA isolated from Colo 320 cells treated with different concentrations of the test compound for 8 h as described in the experimental section. As illustrated in Figure 4B, application of 18.42 µM (7 µg/mL) SF002-96-1 reduced the survivin mRNA level in Colo 320 cells by around 50%. To confirm the data obtained from the qRT-PCR experiments, Western blot experiments were performed for survivin protein expression. As shown in Figure 4C, SF002-96-1 significantly reduced endogenous survivin protein level starting at 13.16 µM (5 µg/mL) after 8 h treatment which suggests that the suppression of survivin by SF002-96-1 is through the transcriptional inhibition of the survivin gene promoter.

**Figure 5:**
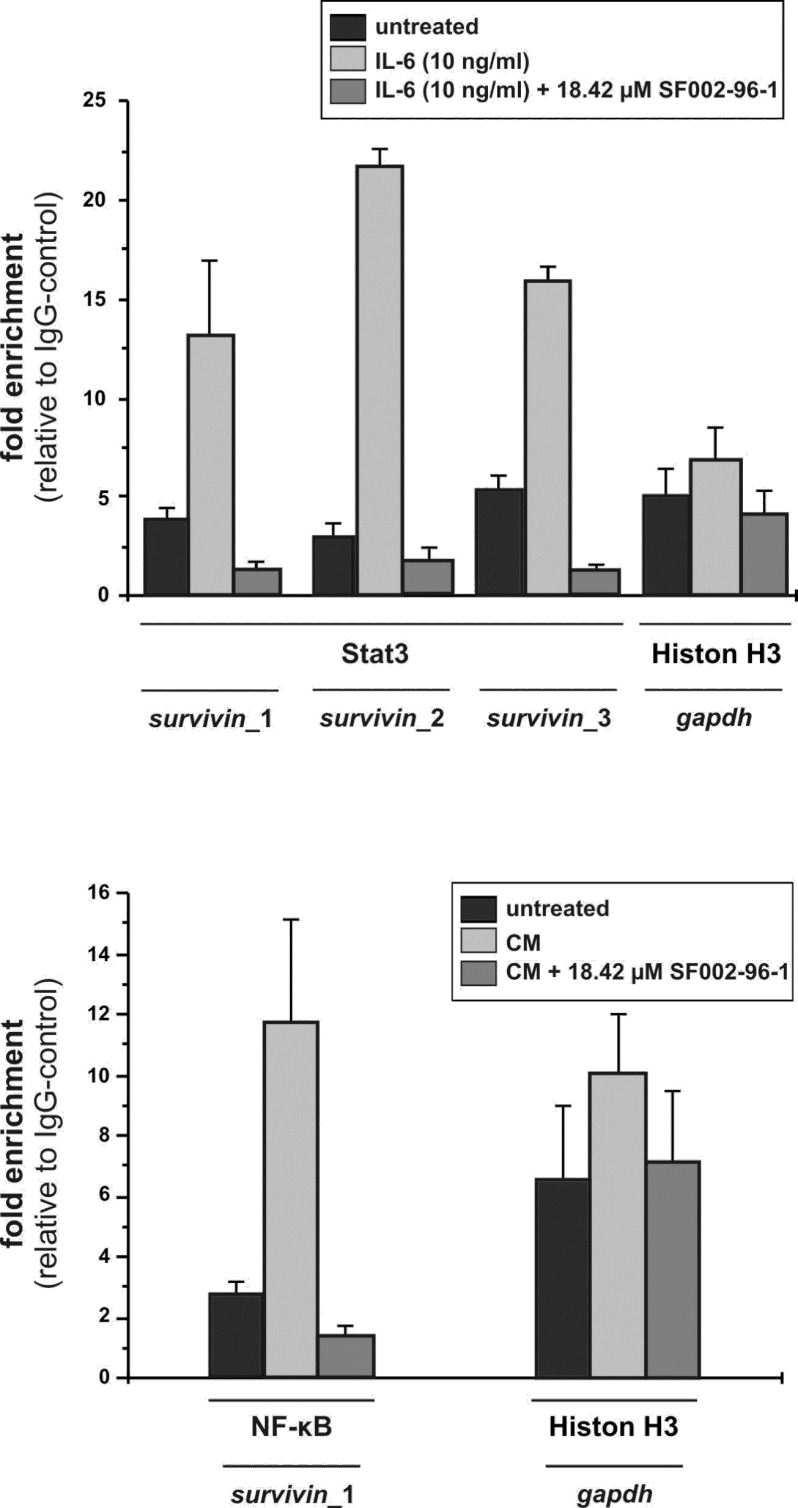
Effect of SF002-96-1 on Sat3 and NF-κB binding to the survivin promoter in Colo 320 cells analyzed by ChIP assay. Colo 320 cells were pretreated with 18.42 µM (5 µg/mL) SF002-96-1 for 1 h before stimulation with 10 ng/mL IL-6 (for Stat3) and 10 ng/mL TNF-α, 5 ng/mL IL-1β (for NF-κB) for 30 min. Chromatin was cross-linked and immunoprecipitated using antibodies against Stat3, NF-κB p65, acetylated histone 3 (H3K9Ac) or rabbit IgG. DNA isolated from immunoprecipitates or from total chromatin preparation before immunoprecipitation (input) was subjected to quantitative real-time PCR using primers specific for Stat3 and p65 binding sites in the survivin promoter or control primers flanking the *gapdh* promoter. Values are expressed as fold enrichment of transcription factor binding relative to IgG as negative control. Data are shown as mean values ± SD of three independent experiments.

To further investigate whether the fungal compound could affect the binding of Stat3 and NF-κB to the survivin promoter in living cells, we performed ChIP assays with primers covering suggested Stat3 and NF-κB (p65) binding sites [23–24]. Q-PCR of the −1231/−1009 (primers Sat3_1), −131/+46 (primers Stat3_2) and −920/−773 (primers Stat3_3) regions of the survivin promoter with Stat3-immunoprecipitated and IL-6 treated samples resulted in a strong induction of Stat3 binding to the two distal as well as to the proximal binding sites of the survivin promoter (Figure 5).

Pretreatment of the cells with 18.42 µM SF002-96-1 resulted in a strong reduction of Stat3 binding to all binding sites in the survivin promoter. ChIP experiments with primers comprising the suggested proximal NF-κB p65 binding site surrounding the transcriptional start site revealed a reduction of p65 binding after stimulation of NF-κB activity with 10 ng/mL TNF-α, 5 ng/mL IL-1β and treatment of the cells with the fungal compound (Figure 5), corroborating the results obtained in the reporter gene assays. As a control, we investigated the influence of the compound on the binding of lysine9 acetylated histone H3 (H3K9Ac) to the constitutive *gapdh* promoter as a marker for accessible chromatin that is transcriptionally active. SF002-96-1 did not significantly affect the levels of H3K9Ac in the constitutive *gpdh* promoter (Figure 5). These results indicate that the compound inhibits survivin expression by preventing the DNA binding of Stat3 and NF-κB transcription factors.

**Figure 6:**
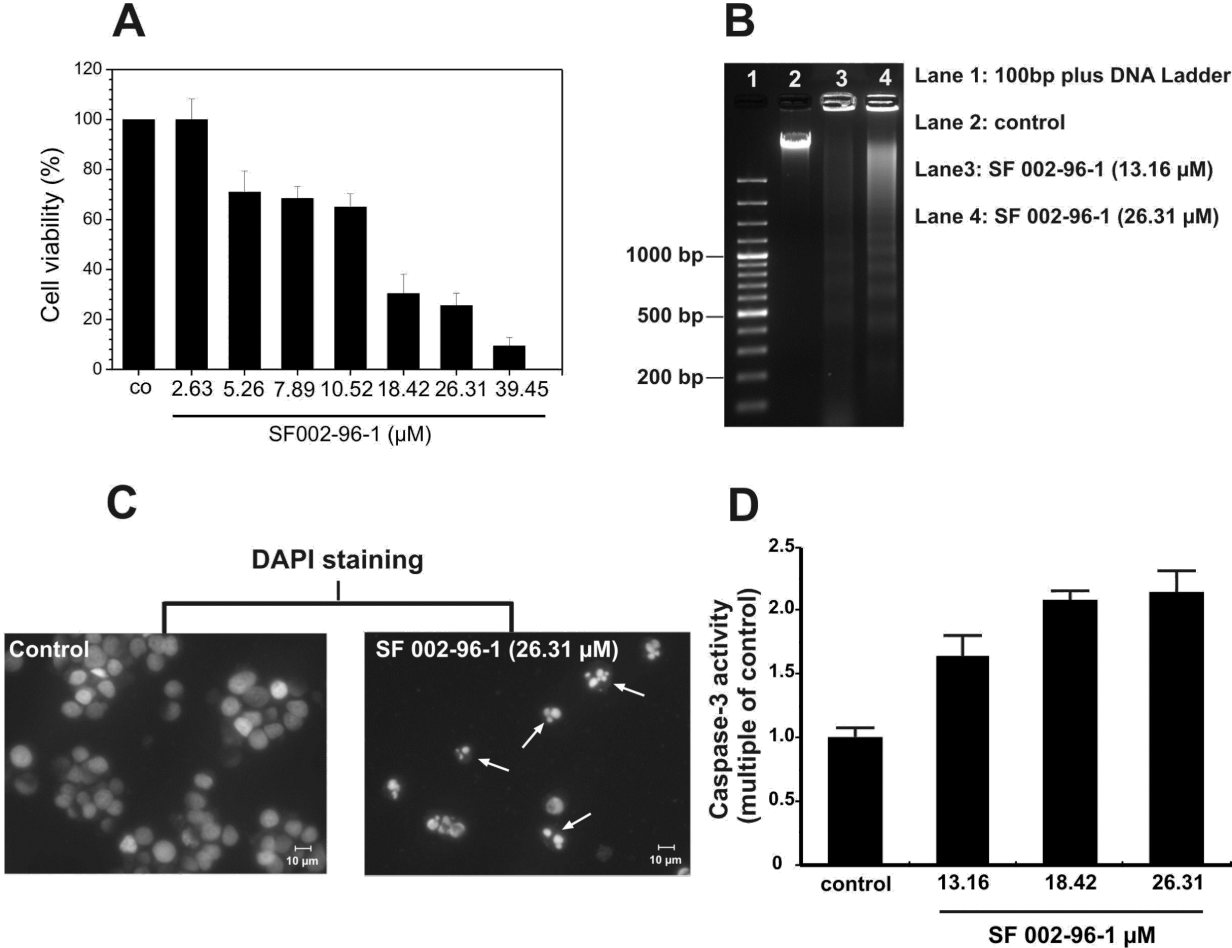
SF002-96-1 induces apoptosis of Colo 320 cells. (A) Colo 320 cells were treated without (control) or with SF002-96-1 at the indicated concentrations for 48 h. Cell viability was determined in triplicate using the XTT colorimetric assay. (B) Detection of DNA fragmentation by agarose gel electrophoresis after treatment of Colo 320 cells for 5 h with different concentrations of the test compound. Control: untreated cells only. (C) Morphology analysis of the cell nuclei by DAPI staining after treatment of Colo 320 cells with 26.31 µM of SF002-96-1 for 5 h. (D) Induction of apoptosis by SF002-96-1 as measured by caspase-3 activity. Colo 320 cells were treated without (control) and with different concentrations of SF002-96-1 for 5 h. After lysis of the cells, 20 µM Ac-DEVD-AMC was added as fluorogenic substrate. AMC released from Ac-DEVD-AMC was measured by a spectrofluorimeter with excitation and emission wavelengths of 335 nm and 460 nm, respectively. Data are shown as mean values ± SD of three independent experiments.

To determine the induction of cell death by SF002-96-1 in Colo 320 cells, the cells were treated with the compound for 48 h, after which the cell viability was assessed by measuring the reduction of the tetrazolium compound 2,3-bis-(2-methoxy-4-nitro-5-sulfophenyl)-2*H*-tetrazolium-5-carboxanilide sodium (XTT) into a colored formazan. SF002-96-1, at concentrations from 10.5 µM (4 µg/mL) significantly decreased cell viability of Colo 320 cells in a dose-dependent manner (Figure 6A). In our studies, the compound proved to be a strong inducer of apoptosis in Colo-320 cells showing the typically biochemical characteristic of apoptosis, like cleavage of the chromosomal DNA at internucleosomal sites into fragments of approximately 200 bp and the fragmented morphology of the nuclear bodies (Figure 6B,C) after 5 h of treatment. Exposure of the cells with different concentrations of SF002-96-1 showed a concomitant increase in caspase-3 activity (Figure 6D), indicating that the compound triggers the apoptotic cascade.

Although several agents including natural products (e.g. curcumin, cucurbitacin, resveratrol) have been described to inhibit survivin expression by interfering with oncogenic signaling pathways [25–27], only two survivin transcriptional inhibitors (e. g. YM155, terameprocol) are in development or in clinical trials [28–29].

Recently, FL188, a camptothecin analog, has been described to inhibit survivin promoter activity and inhibits the expression of cancer-associated survival genes (Mcl-1, XIAP, cAP2) in p53 status independent manner at nanomolar concentrations [30]. We have identified a new drimane sesquiterpene lactone, SF002-96-1, which inhibits survivin expression by interfering with critical signaling cascades (JAK/Stat, NF-κB) involved in transcriptional activation of the survivin promoter and subsequently triggering apoptosis in Colo 320 cells. Drimane sesquiterpenes are widespread in plants, fungi and marine organisms such as algae, sponges and corals and have attracted some attention for their potent antibacterial, antifungal, cytotoxic, antifeedant, phytotoxic, piscicidal and molluscicidal activities [31–33].

## Conclusion

In summary, we identified a new fungal drimane sesquiterpene lactone, SF002-96-1, which inhibits survivin expression by blocking the binding of critical transcription factors (Stat3, NF-κB) to the promoter of the *survivin* gene and triggers apoptosis in the colon carcinoma cell line Colo 320. Due to the lack of a larger portfolio of survivin antagonists, SF002-96-1 may serve as lead structure for the development of novel cancer therapeutics. Further investigations on the cellular targets and the mode of action of the compound are now under way.

## Experimental

### General procedures

1D and 2D NMR data were recorded with a Bruker AVANCE III 600 MHz spectrometer equipped with a 5 mm inverse TCI cryoprobe using standard pulse sequences. APCI–MS spectra were measured from a solution of the analyte in MeCN/H_2_O with a Hewlett Packard MSD 1100 using an evaporator temperature of 400 °C, a drying gas temperature of 350 °C at a flow of 6 L/h (N_2_). In positive ionization mode, the capillary voltage amounted to 3.5 kV, the corona discharge current was 4 μA. In negative ionization mode, the capillary voltage amounted to 2.2 kV, the corona discharge current was 6 μA. HRESI–MS data were measured from a solution of the analyte in acetonitrile with a Waters Q-TOF-Ultima 3 equipped with a LockSpray interface (tri-*n*-octylamine as external reference). IR and UV spectra were measured with a Bruker IFS48 FTIR spectrometer and a Perkin-Elmer Lambda-16 spectrophotometer, respectively. The optical rotation was measured on a Perkin-Elmer 241 polarimeter at 578 nm and 546 nm and extrapolated to 589 nm using Drude’s equation [34].

### Producing organism, fermentation and isolation of compound SF002-96-1

*Aspergillus sp.* strain IBWF002-96 was obtained from the culture collection of the Institute of Biotechnology and Drug Research (IBWF e.V.), Kaiserslautern, Germany. The strain IBWF002-96 showed all characteristics of the genus *Aspergillus*, the species however could not be unequivocally determined. ITS sequence analysis of the ITS1-5.8S rDNA-ITS2 region of nuclear DNA [35] showed high similarity to an uncultured soil fungus (100% in 554 bp, Genbank accession no. GQ921753.1) and to *Aspergillus janus* (98% in 575 bp, Genbank accession no. EU021598).

For maintenance, the fungus was grown on HMG agar slants consisting of: 1% malt extract, 1% glucose, 0.4% yeast extract, pH 5.5 and 2% agar for solid media. Fermentation was performed in a Biolafitte C-6 fermenter containing 20 L of KG media (0.4% mashed potato flakes, 2% glucose, pH 5.5) with aeration (3 L air/min) and agitation (120 rpm) at 22 °C. The production of SF002-96-1 was followed by the inhibitory effect of various concentrations of a crude extract of the culture fluid in the survivin promoter dependent reporter gene assay as described below. The compounds were isolated from the culture fluid by bioactivity-guided fractionation using the survivin transcriptional reporter assay in Colo 320 cells. The fermentation was stopped after 18 days, when the glucose in the medium was depleted and the inhibition of the survivin promoter activity reached a maximum. The culture fluid was separated from the mycelium by filtration, extracted twice with an equal volume of ethyl acetate (EtOAc) and dried over Na_2_SO_4_. The solvent was evaporated in vacuo and the crude extract (3.2 g) was separated by chromatography on silica gel (Merck 60). Elution with cyclohexane/EtOAc (50:50 v/v) resulted in 580 mg of an enriched fraction which was further purified by preparative HPLC (Macherey-Nagel, Düren, Germany; Nucleosil RP18; column 21 × 250 mm, flow 20 mL/min) with MeCN/H_2_O (60:40) as eluent to yield 28 mg of SF002-96-1 (*t*_R_: 13 min). During bioactivity-guided fractionation and isolation of compound SF002-96-1, no other compounds have been detected inhibiting survivin promoter activity.

**Figure 7:**
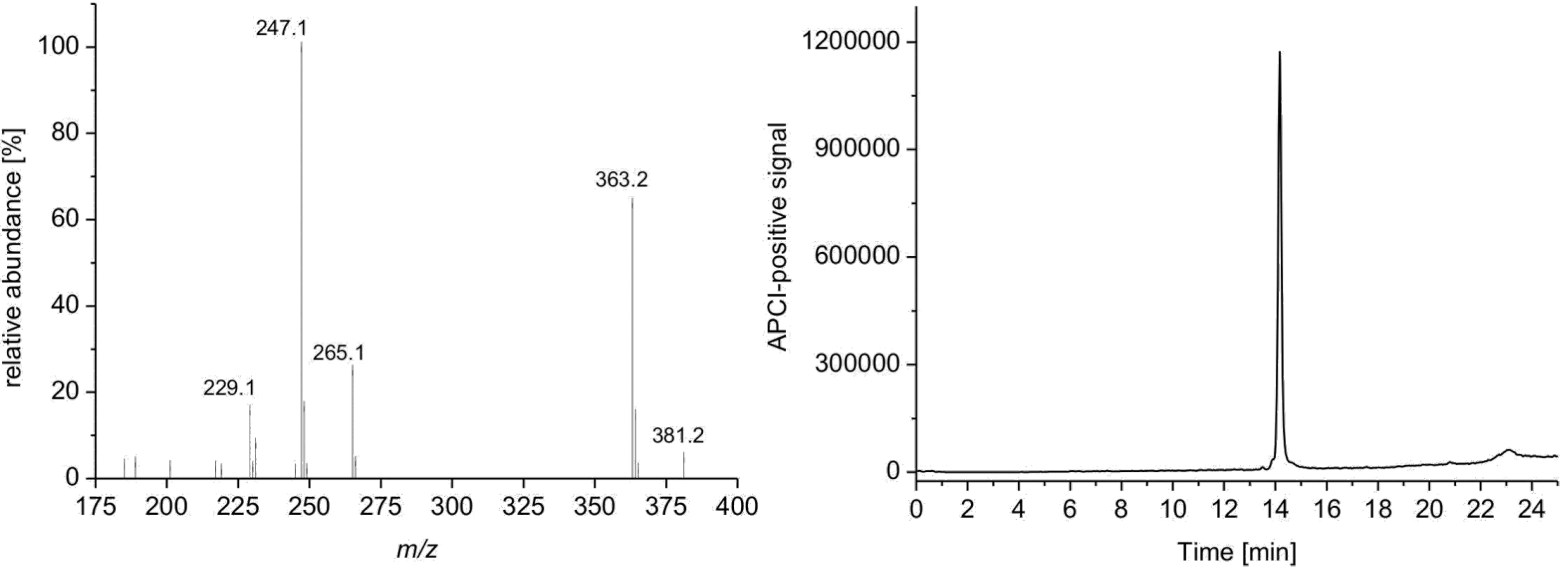
Single-quadrupole mass spectrumof SF002-96-1 obtained using an atmospheric pressure chemical ionization interface showing the pseudo-molecular ion. HPLC–MS analysis of the purified compound (APCI, total ion current).

The purity of the isolated compound was analyzed with a Hewlett-Packard Series 1100LC-MSD instrument ﬁtted with a LiChroCART Superspher 100 RP-18 column (125 × 2 mm, 4 mm particle size; Merck). The chromatographic conditions consisted of a gradient from 1% to 100% acetonitrile in 20 min, and an isocratic step at 100% acetonitrile for 1 min at 40 °C and 10 µL injection volume was used. The ﬂow rate was 0.45 mL/min. The fragmentor voltage was set to 140 V in the positive and negative APCI modes. The compound showed the highly characteristic fragmentation pattern in the APCI-positive mass spectrum revealing the pseudo-molecular ion [M + H]^+^ with *m*/*z* of 281.2. The purity of SF002-96-1 as estimated by HPLC-DAD/MS analysis was greater than 98.5% (Figure 7).

SF002-96-1: colourless oil, UV (CH_3_CN) (λ_max_, log ε): 207 (3.94) nm; [α]_D_^20^ –167.0 (*c* 0.4, CD_3_CN); IR (ν): 3411, 2934, 1757, 1737, 1632, 1464, 1379, 1249 cm^–1^; HRESI–MS (*m*/*z*): [M + Na]^+^ calcd for C_21_H_32_O_6_Na, 403.2097; found, 403.2090; APCI–MS neg. mode *m*/*z*: 380.2 [M]^+^, 362 [M − H_2_O]^+^, pos. mode *m*/*z*: 363.2 [M − H_2_O + H]^+^, 265.1 [M − H_3_C(CH_2_)_4_CO_2_H + H]^+^, 247.1 [M − H_3_C(CH_2_)_4_CO_2_H − H_2_O + H]^+^; ^1^H NMR (600 MHz, CD_3_CN) δ 6.57 (d, 4.0 Hz, 1H, H-7), 5.72 (dd, 4.0, 4.7 Hz, 1H, H-6), 4.48 (d, 10.5 Hz, 1H, H-11b), 4.23 (d, 10.5 Hz, 1H, H-11a), 4.02 (dd, 4.4, 11.9 Hz, 1H, H-1), 3.76 (br s, 1H, OH-9), 2.96 (br s, 1H, OH-1), 2.32 (m, 2H, H-2’), 1.94 (H-5, overlapped with the solvent peak), 1.66 (m, 1H, H-2b), 1.59 (m, 2H, H-3’), 1.57 (m, 1H, H-2a), 1.37 (m, 1H, H-3b), 1.34 (m, 1H, H-3a), 1.30 (m, 2H, H-4’), 1.28 (m, 2H, H-5’), 1.15 (s, 3H, Me-13), 1.04 (s, 3H, Me-15), 0.97 (s, 3H, Me-14), 0.87 (t, 7.0 Hz, 3H, Me-6); ^13^C NMR (150 MHz, CD_3_CN) δ 173.5 (C-1’), 169.8 (C-12), 133.8 (C-8), 133.5 (C-7), 77.4 (C-11), 77.0 (C-9), 70.7 (C-1), 67.0 (C-6), 45.7 (C-5), 44.7 (C-10), 42.7 (C-3), 35.1 (C-2’), 34.0 (C-4), 32.6 (C-14), 31.9 (C-5’), 28.2 (C-2), 25.1 (C-3’), 24.9 (C-13), 23.0 (C-4’), 14.2 (C-6’), 12.8 (C-15).

### Cell culture

Colo 320 (DSMZ ACC 144) cells were maintained in RPMI 1640 medium with 25 mM HEPES buffer and 2 mM L-glutamine, supplemented with 10% fetal calf serum, 100 U/mL penicillin, 100 μg/mL streptomycin at 37 °C and 5% CO_2_.

### Reporter gene assays

The 1092 bp human survivin promoter (region between nucleotide 1821 and nucleotide 2912 within the human survivin gene; GenBank^TM^ accession number U75285) was amplified by polymerase chain reaction from genomic DNA isolated from MonoMac6 cells using oligonucleotides derived from published sequences [36]. The PCR product was cloned into the *Xho*I-*Hind*III site of the promoterless luciferase vector pGL3-basic (Promega, Mannheim, Germany) to generate the hSurvivin-promoter-driven luciferase reporter plasmid (pGL3-hsurvpro). The plasmid pRL-CMV for normalizing transfection efficiency was obtained from Promega (Dual-Luciferase-Reporter-Assay). The NF-κB driven reporter plasmid pNF-κB-Luc was obtained from Clontech (Saint-Germain-en-Laye, France). The IL-6 responsive STAT3-dependent reporter vector pMW-IRF7 has been described previously [37]. TOPFLASH and FOPFLASH luciferase reporters were obtained from Upstate Biotechnology, Inc. (Lake Placid, USA).

Transient transfections of Colo 320 cells were performed by electroporation (BioRad, Gene Pulser) of 1 × 10^7^ cells/mL in 0.4 mL RPMI 1640 medium containing 10% FCS together with 50 µg of the indicated plasmids at 290 V and 975 m. After electroporation, the cells were seeded at 1 × 10^6^ cells/mL in RPMI medium containing 10% FCS in a 24 well plate and allowed to recover for 24 h. For induction of reporter gene expression, the cells were treated with 10 ng/mL TNF-α and 5 ng/mL IL-1β for pNF-κB-Luc and 10 ng/mL IL-6 for pMW-IRF7 with or without test compounds. Luciferase activity was measured 24 h after induction using the Dual-Glo luciferase assay system (Promega, Mannheim, Germany) according to the manufacturer´s instructions with a luminometer.

### Cell viability testing

The cytotoxicity of the compound was determined after 48 h using a XTT-based cell viability assay as previously described by Roehm et al. [38].

### Quantitative real-time polymerase chain reaction analysis (qRT-PCR analysis)

The mRNA expression in human Colo 320 cells was analyzed by two-step real-time RT-PCR as described before [39] with gene-specific primers for human survivin (Genbank Accession NM001168) forward: 5´-ACCAGGTGAGAAGTGAGGGA-3´ and reverse: 5´-AACAGTAGAGGAGCCAGGGA-3´ (size of the PCR product is 309 bp) and *GAPDH* (Genbank Accession M33197) forward: 5’-CCTCCGGGAAACTGTGG-3’ and reverse: 5’-AGTGGGGACACGGAAG-3’ (size of the PCR product is 140 bp). Relative mRNA amounts were determined using the mathematical model for relative quantification in real-time PCR proposed by Pfaffl [40].

### Chromatin immunoprecipitation (ChIP) assay

Colo 320 cells were seeded out in 75 cm^2^ tissue culture flasks at a density of 5 × 10^5^ cells per mL. After 24 h of cultivation, cells were starved for additional 16 h in RPMI 1640 medium with 0.5% FCS. After that, cells were pretreated with SF002-96-1 for 1 h. Stimulation of the NF-κB signaling pathway was performed for 30 min with a cytokine mixture consisting of 10 ng/mL TNF-α and 5 ng/mL IL-1β. Stimulation of the Stat3 signaling pathway was performed for 30 min with 10 ng/mL IL-6. Chromatin immunoprecipitation (ChIP) assays were performed as described by Carey et al. [41], using specific anti-Stat3 (79D7, New England Biolabs, Frankfurt/M), anti-NF-κB p65 (SC-109X, Santa Cruz Biotechnology, Santa Cruz, CA, USA) and anti-lysine9 acetylated histone H3 antibody (9671, New England Biolabs, Frankfurt/M) at dilutions recommended by the manufacturers (1:50). The immunoprecipitates were pelleted and incubated at 65 °C overnight to reverse cross-links. The DNA was extracted by phenol–chloroform extraction and precipitated with ethanol. The precipitated DNA was resuspended in 50 µL TE buffer (10 mM Tris-HCl, 1 mM EDTA, pH 7.4) and used for PCR amplification. Three putative Stat3-binding sites within the survivin promoter were analyzed. The sequences of the PCR primers used are as followed: Stat3_1: forward primer, 5´-CAGTGAGCTGAGATCATGCC-3´; 5´-TATTAGCCCTCCAGCCCCAC-3´ reverse primer (fragment size: 223 bp), Stat3_2: forward primer, 5´-CGCCTCTACTCCCAGAAG-3´; 5´-TGTAGAGATGCGGTGGTC-3´ reverse primer (fragment size: 195 bp), Stat3_3: forward primer, 5´-CCAAAGCAGAGGACACAC-3´; 5´-GGCCACTACCGTGATAAG-3´ reverse primer (fragment size: 165 bp). The primer sequences used for the NF-κB binding site within the survivin promoter were: NF-κB: forward primer, 5´-CTGCACGCGTTCTTTGA-3´; 5´-GCGGTGGTCCTTGAGA-3´ reverse primer (fragment size: 327 bp). The gapdh primer mixture was obtained from New England Biolabs, Frankfurt/M (SimpleChIP^®^ Human GAPDH Exon 1 primers).

### Western immunoblotting

Colo 320 cells were seeded into a 6-well plate at cell density of 5 × 10^5^ cells/mL and allowed to grow for 24 h. The cells were then treated for an additional 8 h with and without different concentrations of test compound and total cell extracts were prepared by lysing the cells with Totex buffer (20 mM HEPES, pH 7.4, 350 mM NaCl, 20% glycerol, 1% NP-40, 1 mM MgCl_2_, 0.5 mM EDTA, 0.1 mM EGTA, 0.5 mM DTT, 10 mM β-glycerophosphate, 10 mM NaF, 1 mM Na_3_VO_4_, 1:25 complete protease-inhibitor cocktail according to the manufacturer´s recommendation (Roche Diagnostics, Mannheim, Germany)). 25 µg total cell extracts were subjected to 10% SDS-PAGE, transferred onto a nitrocellulose membrane, probed with antibodies specific for human survivin (1:1000, 71G4B7, New England Biolabs Frankfurt/M) or β-actin (1:1000, 13E5, New England Biolabs Frankfurt/M) and then with the appropriate secondary antibody conjugated to horse radish peroxidase (1:2000, Anti-rabbit IgG, HRP-linked New England Biolabs, Germany). Immunoreactive proteins were visualized by the enhanced chemoluminiscent detection system (New England Biolabs, Germany).

### Caspase-3 activity assay

Colo 320 cells were seeded at a density of 5 × 10^5^ cells/mL in 96 well plates and treated with test compound for 5 h. After incubation, the cells were centrifuged at 1000*g* for 10 min at 4 °C, washed with PBS and lysed with 50 µL cell lysis buffer (50 mM HEPES, 0.1% CHAPS, 5 mM DTT, 0.1 mM EDTA, pH 7.4). After a freezing step at −80 °C and rethawing, the cell lysate was centrifuged at 1000*g* for 10 min at 4 °C. 25 µL of the supernatants were transferred into a new 96-well plate and incubated with 75 µL assay buffer (50 mM HEPES, 100 mM NaCl, 0.1% CHAPS, 10 mM DTT, 1 mM EDTA, 10% glycerol, pH: 7.4) containing 20 µM Ac-DEVD-amino-4-methylcoumarin (AC-DEVD-AMC; Calbiochem, Bad Soden, Germany) for 2 h at 37 °C. AMC released from the cleavage of AC-DEVD-AMC was measured by a spectrofluorimeter with excitation and emission wavelengths of 355 nm and 460 nm respectively.

### Measurement of DNA fragmentation

Colo 320 cells were resuspended at 5 × 10^6^–1 × 10^7^ cells/mL and incubated with the test compound for 5 h. After incubation, the cells were centrifugated (1000*g*, 10 min), washed once with ice cold PBS and lysed with 600 µL lysis buffer (10 mM Tris-HCl, 10 mM EDTA, 0.2% Triton X-100, pH 7.5) on ice for 10 min. The lysate was centrifuged at 14 000 rpm for 10 min at 4 °C and the supernatant was extracted twice with phenol/chloroform/isoamyl alcohol (25:24:1). The aqueous phase was precipitated with two volumes of ethanol (100%) and 0.1 volume of sodium acetate (3 M, pH 5) overnight. The DNA pellet was rinsed with ethanol (70%) and dissolved in TE buffer (10 mM Tris-HCl, 1 mM EDTA, pH: 7.4) and incubated with RNase (0.1 mg/mL) for 30 min at 37 °C. The samples were separated on a 1.5 % agarose gel and stained with ethidium bromide.

### DAPI staining

Colo 320 cells were incubated for 5 h with test compound and washed once with ice cold PBS. After centrifugation at 1000*g* for 10 min at 4 °C, the cell pellet was resuspend in 5% PBS buffered paraformaldehyde solution containing 10 µg/mL DAPI (4',6-diamidino-2-phenylindole) and incubated for 10 min on ice. A portion (10 µL) of the cell suspension was then placed on a glass-slide, covered with a coverslip and the morphology of the cell nuclei was observed by using a fluorescence microscope (Zeiss, Axioskop).

## Supporting Information

File 1 1D and 2D NMR spectra of compound SF002-96-1.
