# Supplementary material for: SF002-96-1, a new drimane sesquiterpene lactone from an Aspergillus species, inhibits survivin expression
Source: Beilstein J Org Chem. 2013 Dec 13;9:2866–76. doi: 10.3762/bjoc.9.323 (PMC3869210; doi:10.3762/bjoc.9.323)
Supplement: File 1 — 1D and 2D NMR spectra of compound SF002-96-1. [file Beilstein_J_Org_Chem-09-2866-s001.pdf]

SF002-96-1, a new drimane sesquiterpene lactone from an *Aspergillus* species, inhibits survivin expression

Silke Felix[1], Louis P. Sandjo[2], Till Opatz*[,2] and Gerhard Erkel*[,3]

Address: [1]Institute of Biotechnology and Drug Research (IBWF), Erwin-Schrödinger-Straße 56, D-67663 Kaiserslautern, Germany, [2]Institute of Organic Chemistry, University of Mainz, Duesbergweg 10-14, D-55128 Mainz, Germany, and [3]Department of Molecular Biotechnology and Systems Biology, University of Kaiserslautern, Paul-Ehrlich-Straße 23, D-67663 Kaiserslautern, Germany

Email: Till Opatz - opatz@uni-mainz.de; Gerhard Erkel - erkel@bio.uni-kl.de

*Corresponding author

1D and 2D NMR spectra of compound SF002-96-1

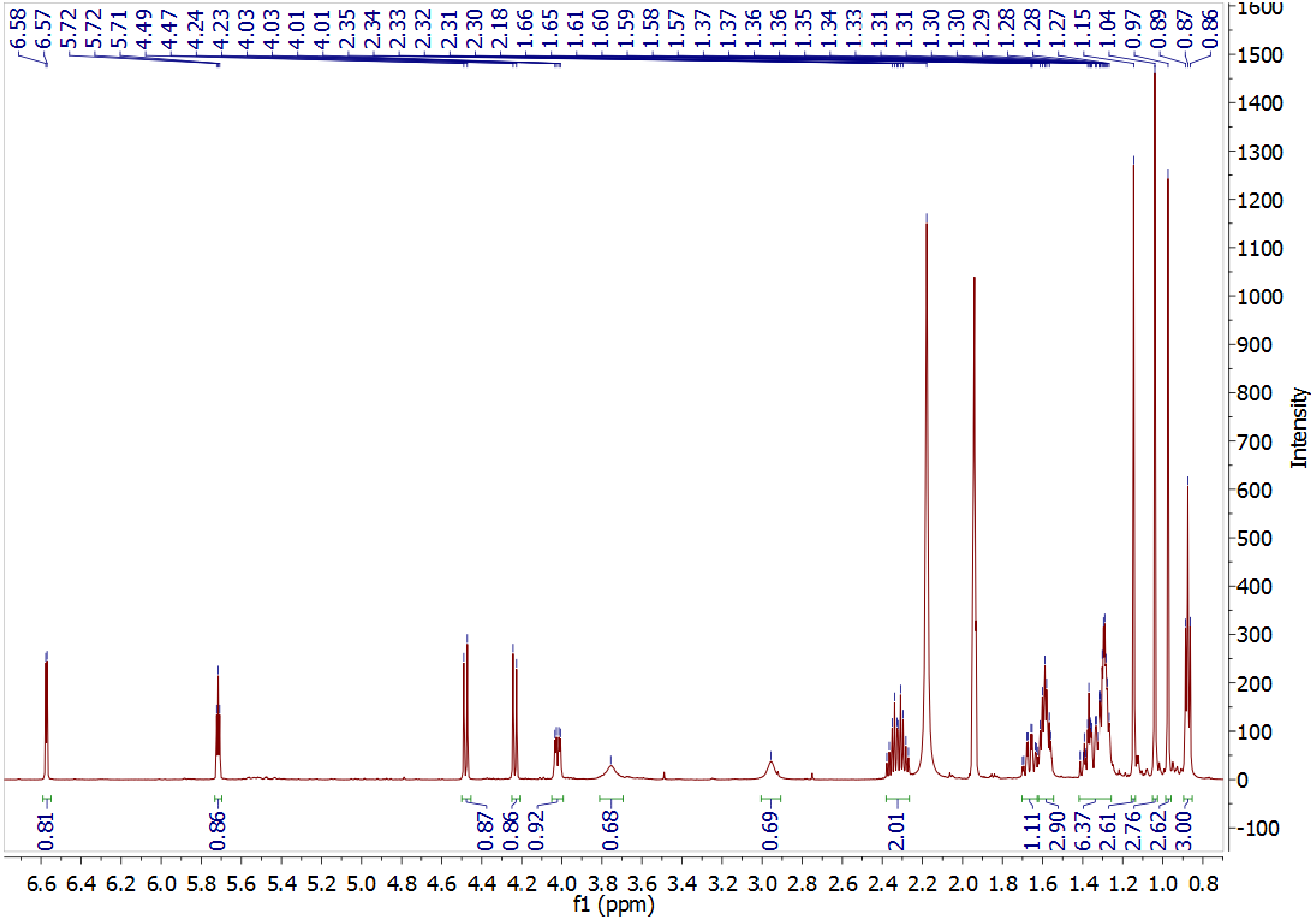

Figure S1: ^{1}H NMR spectrum of compound **1** (CD_3CN).

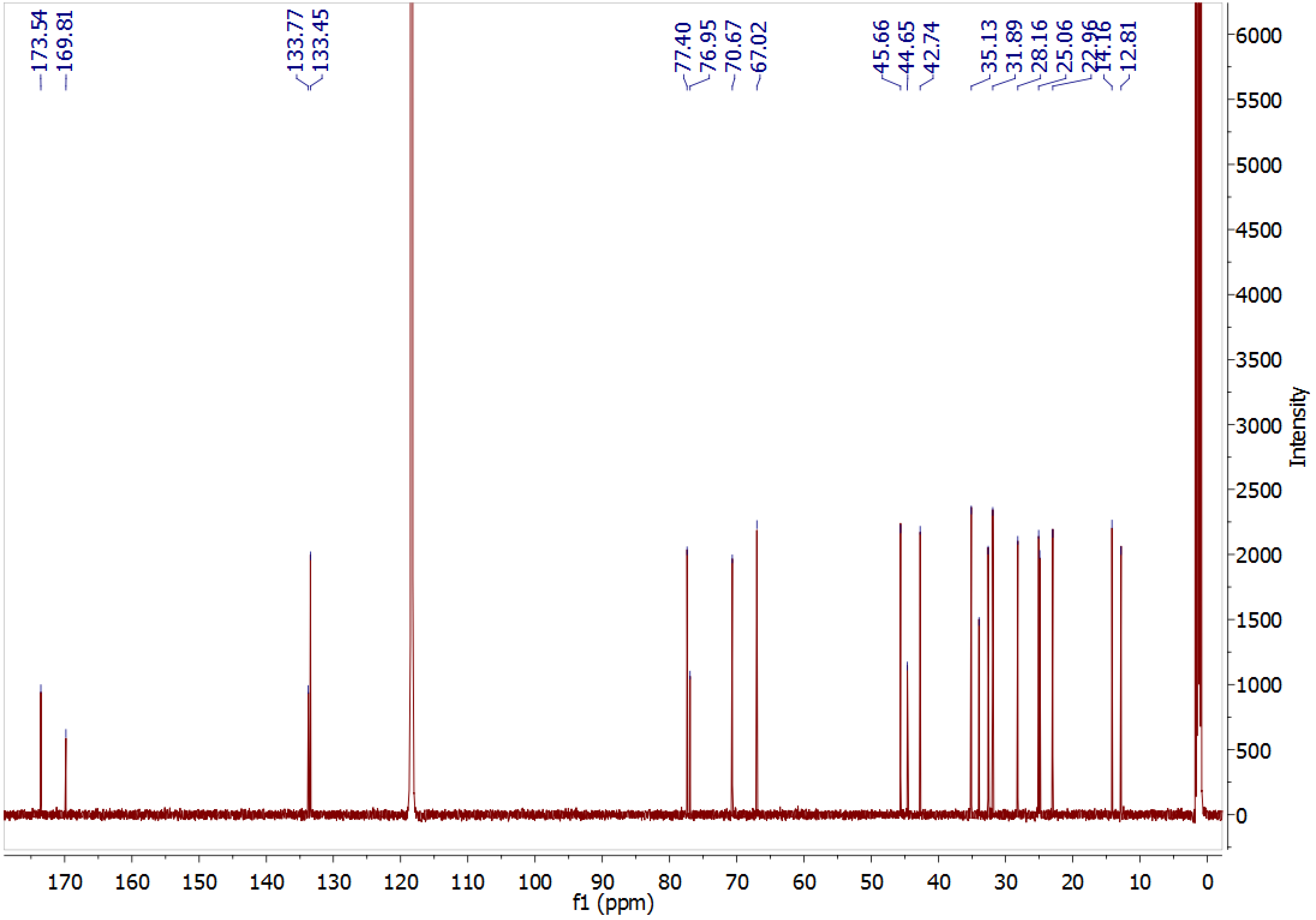

Figure S2: ^{13}C NMR spectrum of compound **1** (CD_3CN).

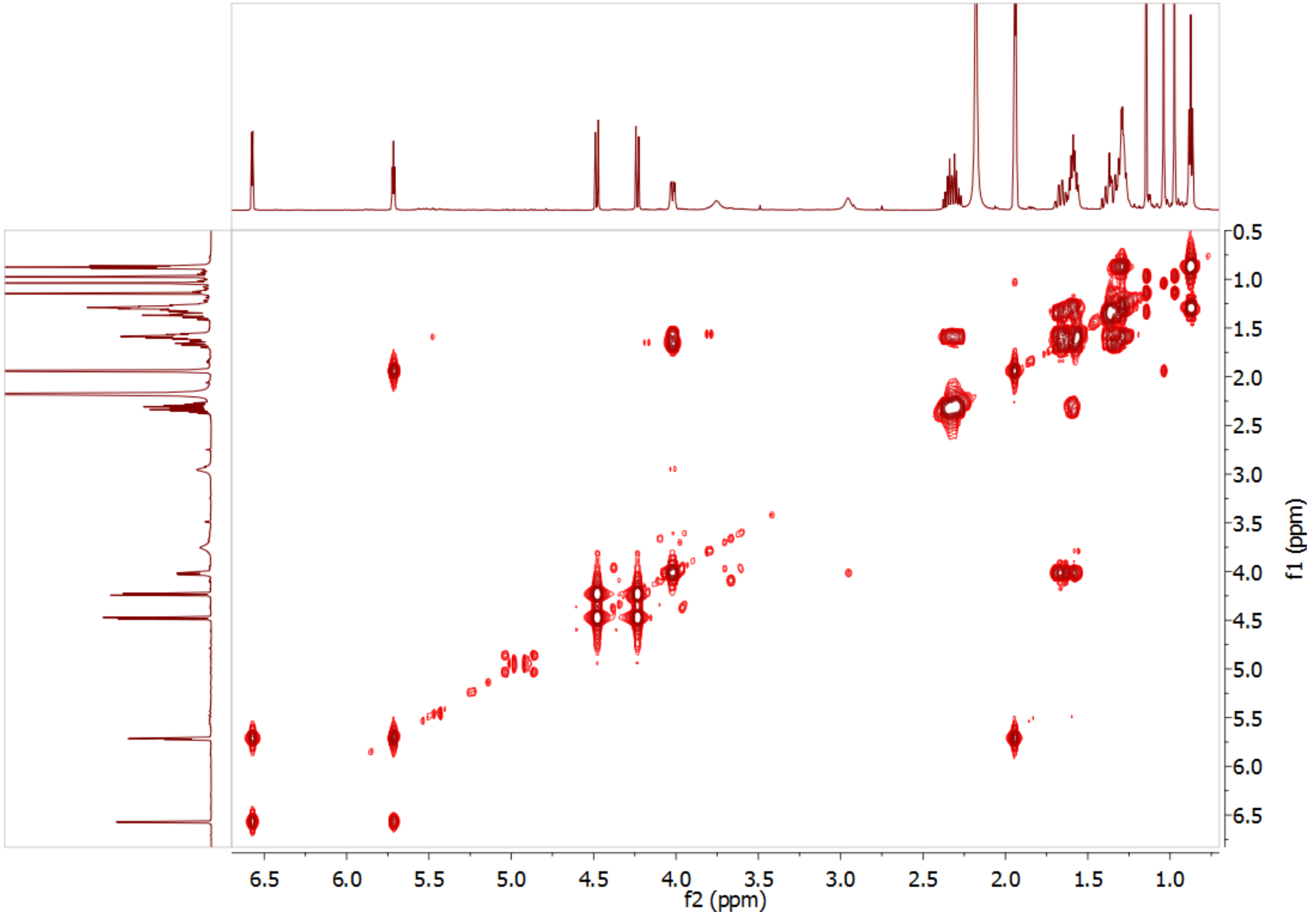

Figure S3: COSY spectrum of compound **1** (CD_3CN).

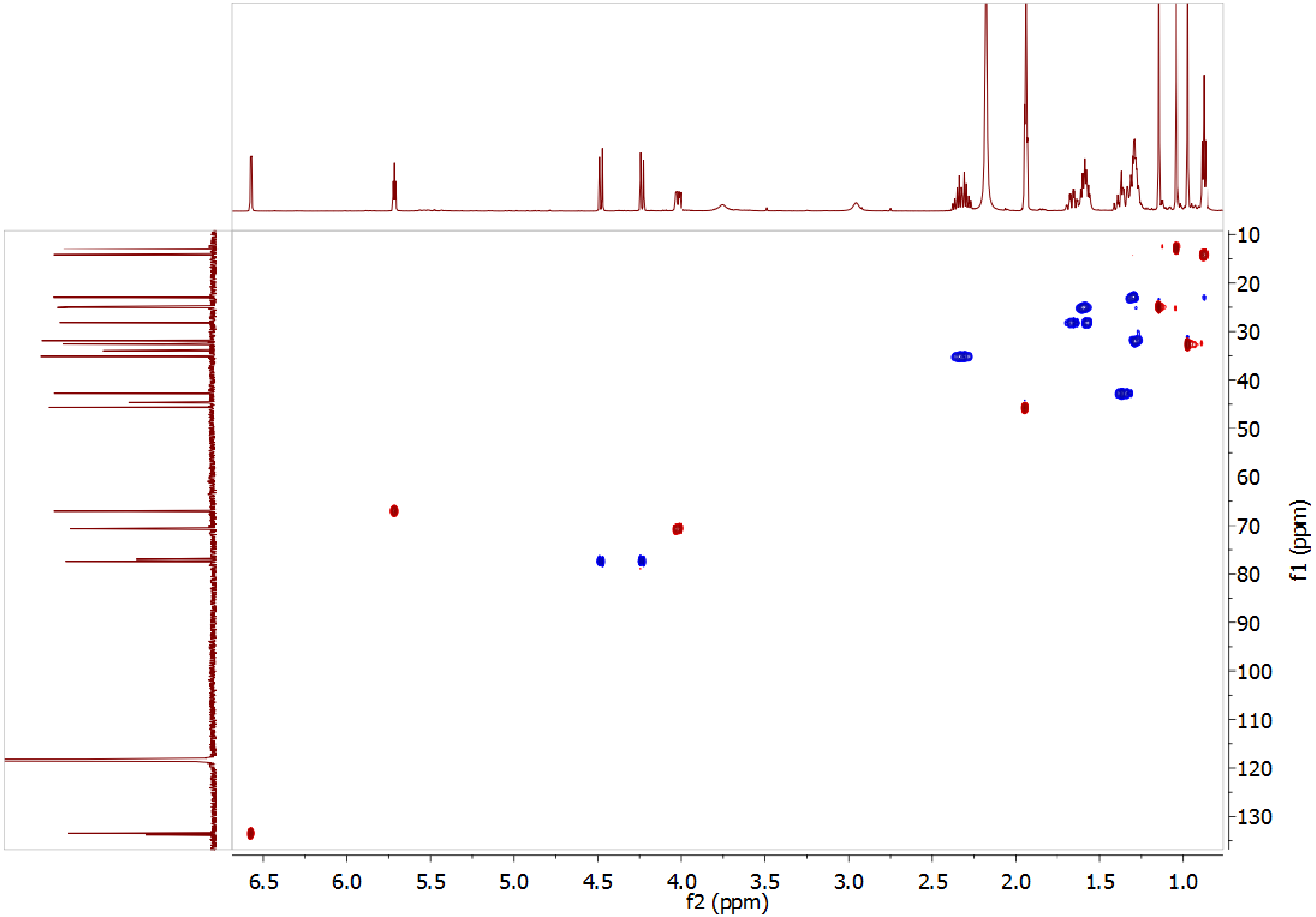

Figure S4: HMQC spectrum of compound **1** (CD_3CN).

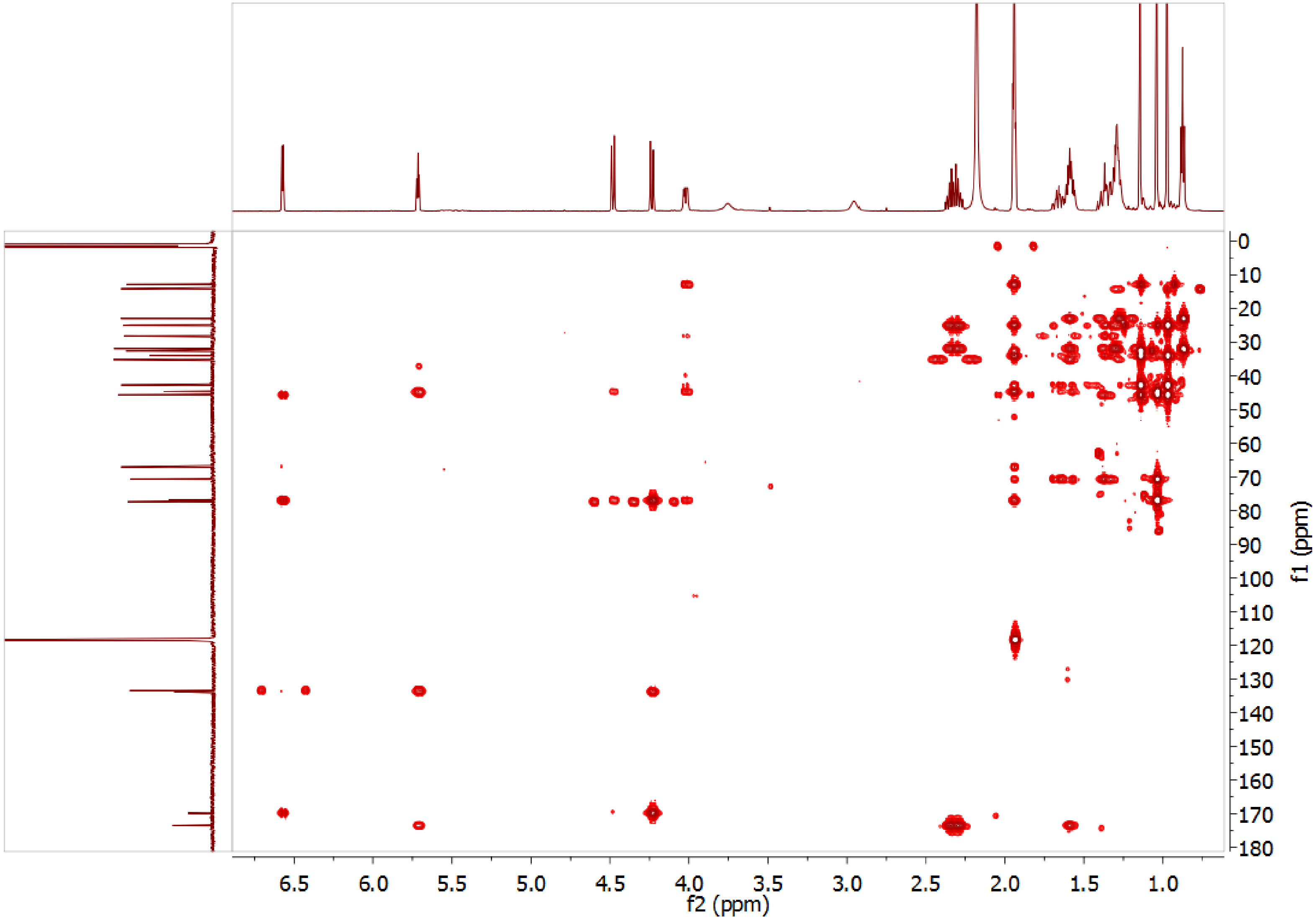

Figure S5: HMBC spectrum of compound **1** (CD_3CN).

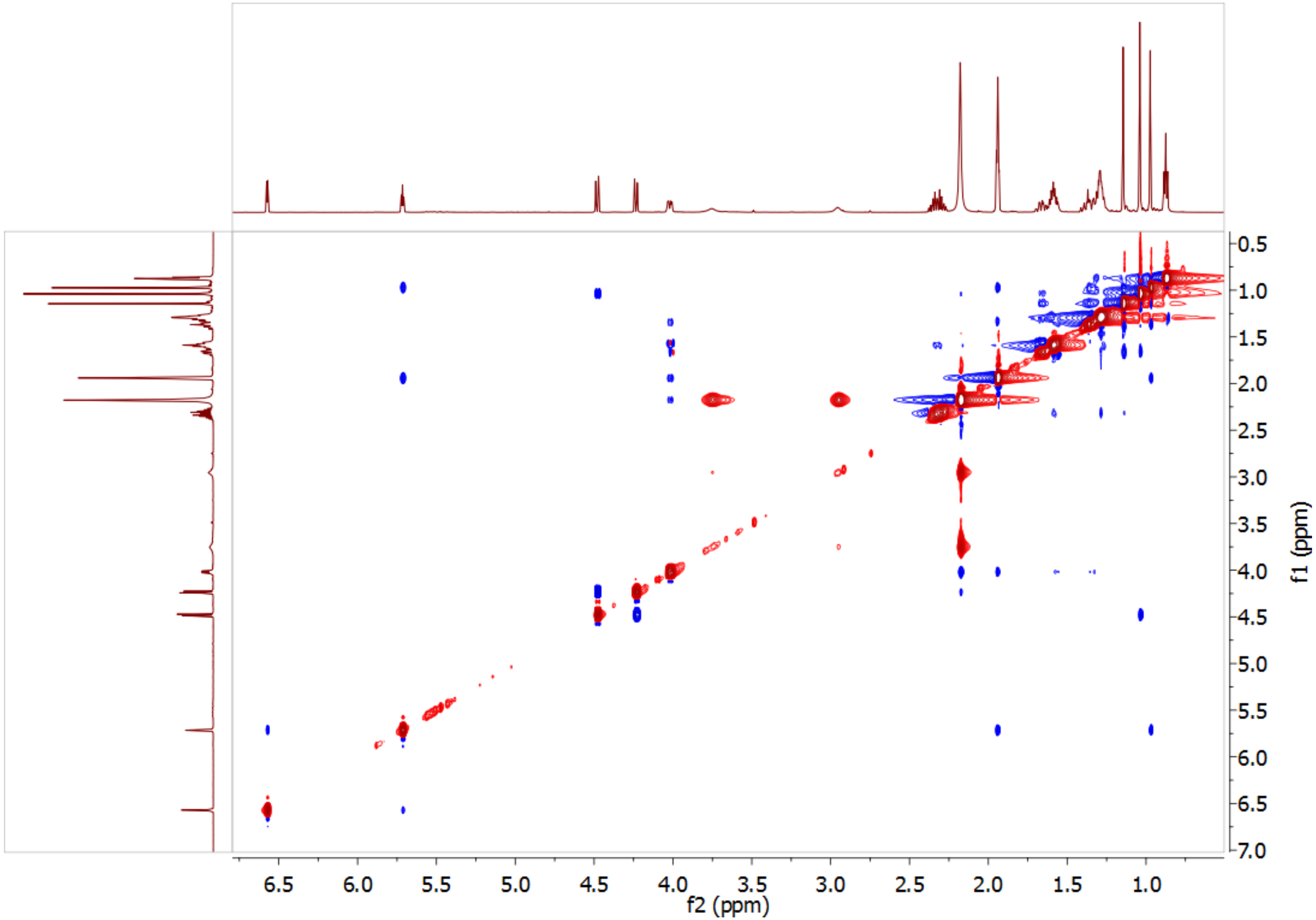

Figure S6: NOESY spectrum of compound **1** (CD_3CN).
